# All-in-one assembly based on 3D-intertangled and cross-jointed architectures of Si/Cu 1D-nanowires for lithium ion batteries

**DOI:** 10.1038/srep08623

**Published:** 2015-02-27

**Authors:** Chihyun Hwang, Tae-Hee Kim, Yoon-Gyo Cho, Jieun Kim, Hyun-Kon Song

**Affiliations:** 1 School of Energy and Chemical Engineering, Ulsan National Institute of Science and Technology (UNIST), Ulsan 689-798, Republic of Korea

## Abstract

All-in-one assemblies of separator, electrode and current collector (SECA) for lithium ion batteries are presented by using 1D nanowires of Si and Cu (nwSi and nwCu). Even without binders, integrity of SECA is secured via structural joints based on ductility of Cu as well as entanglement of nwSi and nwCu. By controlling the ratio of the nanowires, the number of contact points and voids accommodating volume expansion of Si active material are tunable. Zero volume expansion and high energy density are simultaneously achievable by the architecture.

We undo entangled lines of our earphone before connecting it to cell phones. The inter-tangled bundles of hairs are easily found on mesh drain traps or at the corners of dirty rooms. High-aspect-ratio structures are easily inter-tangled with high probability, forming 3D network occupying a volumetric space with voids within its structure as well as keeping integrity of the entangled object bodies. Here, we demonstrate 3D network electrodes of binary phases as anodes for lithium ion batteries (LIBs). The architecture was built by inter-tangling silicon nanowires (nwSi) as an active material with copper nanowires (nwCu) as a conductive pathway. Polymeric binders used in conventional LIBs were not used in this architecture because nwCu plays a role as the binder due to its ductile properties. Structural joints are developed by pressure in an inter-locking way that nwSi is fitted into concaved parts of nwCu. As the second point to distinguish it from conventional electrodes, the 3D architecture was built not on current collectors but on separators. Additional layer of nwCu stacked on bare face of the 3D architecture worked as an ultra-light porous current collector. The separator-electrode-current collector assembly (SECA) showed very superior performances in terms of cyclability and rate capability.

Electrochemical reactions, the potential of which is relatively negative enough to be close to that of Li^+^/Li, have been used as the chemistry for anodes of LIBs, including intercalation (graphites^1^,^2^), conversion reactions (metal oxides^3^,^4^) and alloying reactions (silicon^5^ and tin^6^). As higher energy densities are more emphasized with electric vehicles and stationary energy storage systems rising, high-capacity anode materials such as silicon are attracting interests from academic and industrial societies. Its theoretical capacity approaches ~4,000 mAh g^−1^ at room temperature that is ten times as large as that of widely used graphites (372 mAh g^−1^). However, serious drawbacks difficult to overcome come with the silicon anode materials, including not only low electric conductivity of silicon but also large volume change during lithiation up to ~400 % leading to pulverization of particles. Resultantly, serious decay of capacity with repeated cycles of charging/discharging follows with silicon^7^,^8^,^9^,^10^,^11^. To accommodate the volume expansion of silicon for blocking pulverization, the buffer space was introduced^12^,^13^,^14^,^15^,^16^,^17^,^18^,^19^,^20^ into silicon particles to form porous or hollow structures. Dead mass of silicon pulverized from particles after expansion was designed^17^,^19^,^21^,^22^,^23^,^24^,^25^ to keep integrity to electric pathways by coating the original particles of silicon with conductive materials such as carbon and conducting polymers. Different from the strategies to modify silicon particles, silicon nanowires were grown directly on stainless steel current collectors via vapor-liquid-solid methods or chemical-vapor-deposition methods^26^,^27^,^28^. Enhanced retention of capacity were guaranteed by efficient electron transport along the 1D structure, strain relaxation through the lateral sides of the nanowires and good contact between roots of the nanowires and current collectors. The similar results were achieved^24^ by spray-coating silicon nanowires on carbon textile matrix without binders. The 1D/1D contact between silicon nanowires and conductive materials was emphasized^19^ for good rate capability and remarkable cycling stability. Various works based on silicon 1D structures were compared with our work in terms of electrochemical performances (Table S1)^29^,^30^,^31^,^32^,^33^,^34^.

Based on the merits of 1D structures of nwSi and inter-tangled networking between nwSi and nwCu, we developed all-in-one architectures assembling electrodes and current collectors onto separators (SECA = separator-electrode-current collector assembly) (Figure 1a). An ethanol-based mixture of nwSi and nwCu was vacuum-filtered through a conventionally used polyethylene separator to form a mixed-nanowire electrode layer on the separator. Here, nwSi and nwCu works as an active material and a conducting agent. Then, only-nwCu layer was laminated as a current collector on the electrode layer by using the same filtration. Finally, the tri-layered laminates were dried and pressed to inter-locking structure between nwSi and nwCu in the electrode layer or between nwCu in the current collector layer or between the electrode layer and the current collector layer. The schematic of cross-sectional view (Figure 1b) is realized in the resultant SECA as shown in electron-microscopic images (Figure 1c, Figure S1 and S2) with inter-tangled nanowires confirmed. Line mapping of Si and Cu elements identified each layer consisting of Cu-dominant phase (I) and relatively Si-rich phase (II).

As the first merit achieved by SECA, higher specific and volumetric energy density of cells can be obtained by using small amount of current collectors as well as by eliminating binders. In the conventional tri-component (active material, binder and conducting agent) electrode of this work, copper foils of micrometer thickness (e.g. 18 um thick with 16 mg cm^−2^) are used. The value of thickness occupies 53 % of overall thickness of electrode and 94 % of total mass of electrode, when considering that electrode mass consisting of silicon, binder and conducting agents at 6:2:2 wt. ratio on current collector are 34 um thick with 17 mg cm^−2^. In our SECA of 30 % nwSi as a representative case, however, only 44 % of electrode thickness (14 um/32 um) and 39 % of total mass (1.3 mg cm^−2^/3.3 mg cm^−2^) were occupied by the current collectors of inter-tangled nwCu. Resultantly, for example, lighter electrodes including current collector can be possible by the SECA with only 30 % to 70 % of mass of a conventional tri-component electrode at the same silicon loading of 0.6 mg cm^−2^ (Figure 1d; detailed calculation in supporting information).

The number of contact points between nwSi and nwCu (n_c_) as well as the contact-to-contact distance (L_cc_) totally depends on the nwSi contents (x_Si_) in the nwSi/nwCu composites. Based on the lines-on-a-plate model in which nanowires of the minor component is evenly distributed on the plate consisting of nanowires of the major component, the maximum values of n_c_ (n_c_^max^) and the minimum values of L_cc_ (L_cc_^min^) were calculated as functions of x_Si_ (Figure 1e and f; detailed calculation in supporting information). The n_c_^max^ per total electrode mass (m_tot_) was maximized at the optimized composition x_Si_^opti^ (open circles in e and f) where the contactable area is matched between nwSi and nwCu. The increase in nwSi contents directly leads to the increase in n_c_^max^/m_tot_ up to the optimized composition because nwCu can provide enough contactable faces to nwSi. At nwSi concentrations higher than x_Si_^opti^, the lack of nwCu is experienced by nwSi so that the contact number decreases. From the viewpoint of the contact-to-contact distance, the values of L_cc_^min^ are equivalent to the diameter of nwCu, the smallest value at which a contact point can approach its nearest contact point, at compositions lower than x_Si_^opti^. After the composition, the contact points move further away from their neighborhoods as silicon contents increase and the lack of nwCu become more serious. The composition at x_Si_^opti^ would be the best choice when considering that higher nwSi contents are required with a closer contact. The x_Si_^opti^ can be traced with the radius ratio of r_Cu_/r_Si_ as an independent variable (dashed red lines with red arrows indicating the increasing direction of r_Cu_/r_Si_ in Figure 1e and f): x_Si_^opti^ = 1/(1 + (d_Cu_/d_Si_)(r_Cu_/r_Si_)) with d = density. Relatively thinner nwCu leading to smaller values of r_Cu_/r_Si_ extends the region of the smallest L_cc_^min^ to higher nwSi composition: e.g. x_Si_^opti^ was shifted from 9.3 % at r_Cu_/r_Si_ = 2.5 to 20 % at r_Cu_/r_Si_ = 1.0. Also, the thinner nwCu provides more number of contacts with shorter distance between contacts at the optimized composition.

By using nwCu as a conducting agent as well as a binder, the inter-tangled SECA was successfully developed, showing a stable physical integrity in terms of cohesion between particles and adhesion to separator (Figure 2a). Even when the composites were highly curved, there was no significant deterioration of their integrity observed. Such a good mechanical properties of SECA originates from development of inter-locking joints between nwSi and nwCu due to the ductile nature of copper (Figure 2b). nwCu was squashed by a pestle into flakes stick to a mortar or kneaded to a monolithic lump (photos in Figure 2b). Due to the ductility of copper, structural joints as the second merit of SECA are expected to be developed in the composites of nwSi with nwCu by pressure in an inter-locking way (Figure 2c). Concaved dents are formed in nwCu at contacts with nwSi (or other nwCu) by using the nwSi (or nwCu) as a mechanically hard template. Resultantly, nwCu grabs nwSi or other nwCu at the locking joints. The inter-locking integrity of SECA accommodated stretching strain impressively. Without pressing, SECA did not maintain its integrity under a stress causing the strain at 40 % (-Press in Figure 2d), showing very poor cyclability (black symbols in Figure 2e). After pressing, however, the inter-locking joints between nwSi and nwCu are developed so that the physical integrity of SECA under stretching and higher capacities were guaranteed (+Press in Figure 2d; red line in Figure 2e). Even after cycling change and discharge 100 times, the integrity of SECA was not damaged (Figure S3). Also, the charge transfer resistance (R_CT_, Figure S4) and sheet resistance (R_Sheet_, measured by four probe conductivity measurement) of the SECA decreased after pressing: R_CT_ from 30 ohm to 9 ohm; R_sheet_ from the very high value that is not measured to 50 ohm sq^−1^.

Cells were galvanostatically lithiated/delithiated at 0.2C/0.5C, respectively. The capacities (Q) were calculated based on the mass of silicon. Electrochemical performances were characterized in terms of rate capability and cyclability. Coin half cells were constructed with the SECA and lithium metal. For comparison, a conventional electrode consisting of nwSi, a binder (a mixture of poly(acrylic acid) and sodium carboxymethyl cellulose or PAA/CMC) and carbon black (Super P) at weight ratio of 6:2:2 was tested at the same condition^35^. The amount of nwSi loading was fixed at 0.6 to 0.8 mg cm^−2^ for all samples for fair comparison. In terms of specific capacity normalized by silicon weight (Q/m_Si_ with Q = capacity and m_Si_ = nwSi mass), as expected, the SECA of higher nwCu contents showed higher capacities with better rate capability due to the improvement of electric conduction by nwCu (Figure 3a and b). In the rate capability curve, the conventional electrode of 60 % nwSi as a control was found between SECAs of 50 % nwSi and 70 % nwSi. The capacity retention with cycles (Figure 3d) was comparable between the SECA of 30 % nwSi and the control, showing similarly shaped traces (Figure 3e). The SECA of 50 % and 70 % nwSi was inferior to the SECA of 30 % nwSi due to the lack of nwCu contents. The decay of capacity with cycles was not in a negligible level. However, it should be notified that the decayed capacity with cycles is not due to the electrode structure (SECA or conventional electrodes) but due to the electrochemical nature of nwSi used in our study (e.g., intrinsic pulverization of nwSi^36^). The inference comes from the fact that the similar or almost identical cyclability except of constant capacity gap was obtained with the SECA of 30 % nwSi and the conventional electrode (Figure 3e), both of which provide ideal environments of conduction pathways to nwSi mass.

To emphasize the merits of SECA over the conventional electrode, the rate capability and cyclability data were re-plotted by using the specific capacity normalized by electrode mass including both nwSi composites with other components and current collector (Q/m_Ed + CC_ with m_Ed + CC_ = the mass of electrode (Ed) + current collector (CC)) (Figure 3c and f). By saving the mass of current collector, the capacities of SECA are significantly superior to that of the conventional electrode. Therefore, the benefit achieved by our SECA makes high-energy density cells feasible (Figure S5).

The electrochemical performances of the SECA showing improved capacities results from the combination of its structural merits. Two points have been discussed above. Inter-locking joints between nwSi and nwCu (or nwCu and nwCu) possibly improve physical integrity without binders, accelerate charge transfer between electroactive mass (nwSi) and electric pathways (nwCu), percolate networking of electric conduction pathways and provide mechanical framework to suppress volume expansion of silicon. Ultra-light porous current collector enables high energy density cells by reducing total mass with keeping the electroactive mass. As the third benefit of the SECA structure, in addition, volume expansion of silicon was successfully accommodated by voids of the inter-tangled network of nwSi and nwCu (Figure 4). The conventional tri-component electrode experienced severe volume expansion around 300 % (estimated by thickness change from 15 um to 43 um; Figure 4a and b). However, there were no significant volume change observed with our SECA (Figure 4c and d), even if the thickness of only nwSi/nwCu composite part was difficult to be identified from that of the nwCu current collector after cycling. It was clearly observed that the expanded volume of silicon filled the void between nwCu completely after cycling (Figure 4e and f). Based on a simple model of cross-oriented stacks of nanowires (supporting information), volume fraction of available voids was compared between the SECA and the conventional electrode. Void between nwSi was estimated at 21.5 % of total volume when the nwSi are closely stacked in a configuration where longitudinal direction of aligned-nanowire stacks is 90°-oriented alternatively. The additional gap at 22 nm between nwSi (radius = 20 nm) is required to involve 20 wt. % binder and 20 wt. % conducting agents. In this case, however, there is no void fraction to accommodate the volume expansion of silicon. With the gap at 68.2 nm, void volume is generated enough to compensate 300 % volume expansion. The resultant silicon loading density of the conventional electrode configuration with enough void is calculated at 0.12 g cm^−3^. On the contrary, the SECA of 30 % nwSi had 64.2 % void volume without additional gap required, showing capability of accommodating 280 % volume expansion (355 % and 402 % accommodated by the SECA of 50 % and 70 % nwSi, respectively). The density of silicon loading was estimated at 0.52 g cm^−3^, which is significantly higher than that of the conventional electrode with enough void fraction (0.12 g cm^−3^).

In summary, 3D structures based on inter-tangling of two different nanowires (nwSi and nwCu) was developed in this work to achieve high-energy-density lithium ion batteries. Based on the nwSi/nwCu composites, uniquely designed electrode assemblies (SECA) unifying separator, active electrode part and current collector into one compartment were constructed (Figure S6). The three characteristics are emphasized: (1) inter-locking joints based on ductility of copper enabling binder-free electrode systems, (2) ultra-light porous current collectors enabling high energy densities and (3) optimized void fraction to accommodate volume expansion of silicon. With enhanced rate capability (Q_15C_ = 67.3 % of Q_0.5C_ for SECA with Q_#C_ = capacity at indicated C rate; cf. Q_15C_ = 14.1 % of Q_0.5C_ for a conventional electrode system) and cyclability (Q at the 100^th^ cycle = 71.4 % of Q at the initial cycle for SECA versus 55.7 % for the conventional), the specific capacity normalized by the weight including electrode and current collector was estimated at 350 to 570 mAh g^−1^ which is incomparable with that of the conventional electrode system (45 mAh g^−1^). Also, we believe that the 3D structure can be used as a platform to estimate the volume expansion of silicon material as shown in structure-related modeling.

## Methods

### SECA

10 mg of nwSi (diameter (D) = 40 nm, length (L) = 1 to 20 um; Sigma; Figure S7) and nwCu (D = 100 ~ 200 nm, L = 0.8 to 6 um, ACS material; Figure S7) with a defined ratio was well dispersed in 20 ml ethanol by ultrasonicator at 25°C for 30 min. The mixture was filtrated through a separator (polyethylene; Asahi NH716; Figure S8) by vacuum. Then, porous current-collector layer was laminated on the pre-developed active layer by filtrating 20 ml ethanolic solution of 10 mg nwCu in the same way. The SECA was dried in vacuum oven at 70°C for 3 h to remove ethanol and then mechanically pressed with 12 MPa by oil-hydraulic press.

### Conventional electrode

An aqueous mixture slurry of nwSi, a binder and a conducting agent at 6:2:2 by weight was pasted in 15 um thickness on a current collector (18 um thick Cu foil) by doctor blade. The electrode was dried in vacuum at 150°C for 2 h. A mixture of poly(acrylic acid) (PAA, M_w_ = 3,000,000, Sigma) and sodium carboxymethyl cellulose (CMC, M_w_ = 250,000, Sigma) at a ratio of 1:1 by weight was used as the binder. Carbon black (Super P, TIMCAL) was used as the conducting agent. The loading amount of the active material was around 0.6 mg cm^−2^.

## Author Contributions

C.H. and H.-K.S. conceived the idea and designed experiments. C.H. fabricated electrodes. C.H., T.-H.K., Y.-G.C. and J.K. carried out electrochemical characterization. C.H., T.-H.K. and J.K. performed morphological characterization. C.H. and H.-K.S. analyzed data and co-wrote the paper. H.-K.S. led overall project.

## Supplementary Material

Supplementary Information supporting information

## Figures and Tables

**Figure 1:**
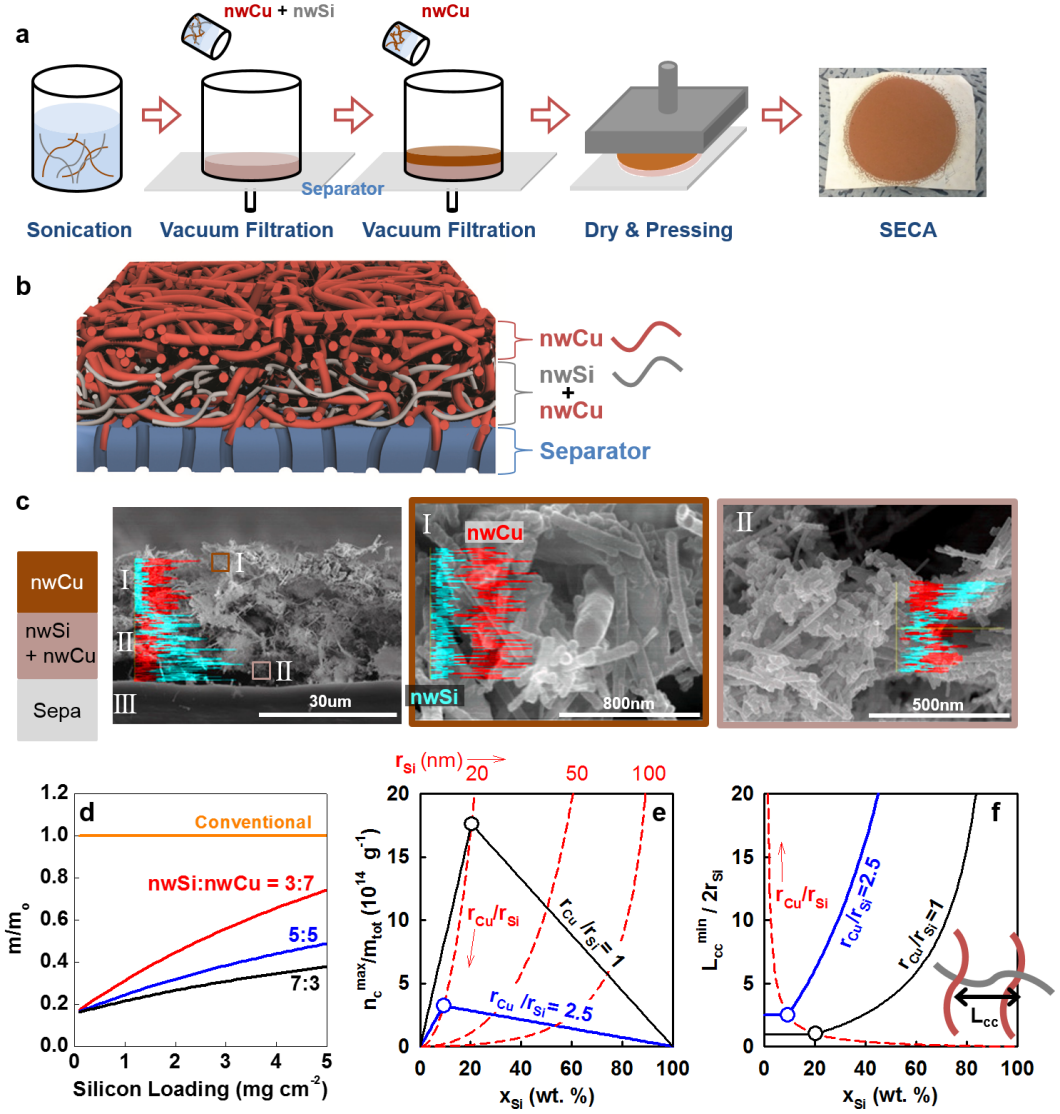
Structure of SECA. (a) Fabrication process of SECA. (b) Schematic cross-sectional view illustrating inter-tangled nanowires in SECA. (c) SEM images of cross-section of SECA with I = current-collector layer, II = electrode layer and III = separator. A portion of I and II indicated by squares in the left photo were magnified in the middle and the right photos. Line mappings of Cu (red) and Si (blue) by energy dispersive spectroscopy were included in each photo. (d) The comparison between SECA and a conventional tri-component electrode in terms of total electrode mass including current collector except of separator. The conventional electrode on 18 um-thick current collector consisted of silicon, binder and carbon black in the weight-based composition at 6:2:2. Three different compositions of SECA were used as indicated by nwSi: nwCu in weight ratio. The relative mass of electrodes (m/m^o^ with m = mass of the sample of interest and m^o^ = mass of the conventional electrode) was used as the ordinate, depending on silicon loading. (e) Dependency of maximum number of contacts between nwSi and nwCu (n_c_^max^) per total mass (m_tot_) of the nwSi/nwCu composites on nwSi contents (x_Si_) in the composites. Radius of nwSi (r_Si_) = 20 nm. (f) Dependency of minimum contact-to-contact distance (L_cc_^min^) on nwSi contents (x_Si_) in the nwSi/nwCu composites. The ratio of L_cc_^min^ to diameter of nwSi was used as the ordinate without dimension, which is the function of only the ratio of diameters (or radius) of nwCu to nwSi at fixed x_Si_ (not the function of absolute values of radii).

**Figure 2:**
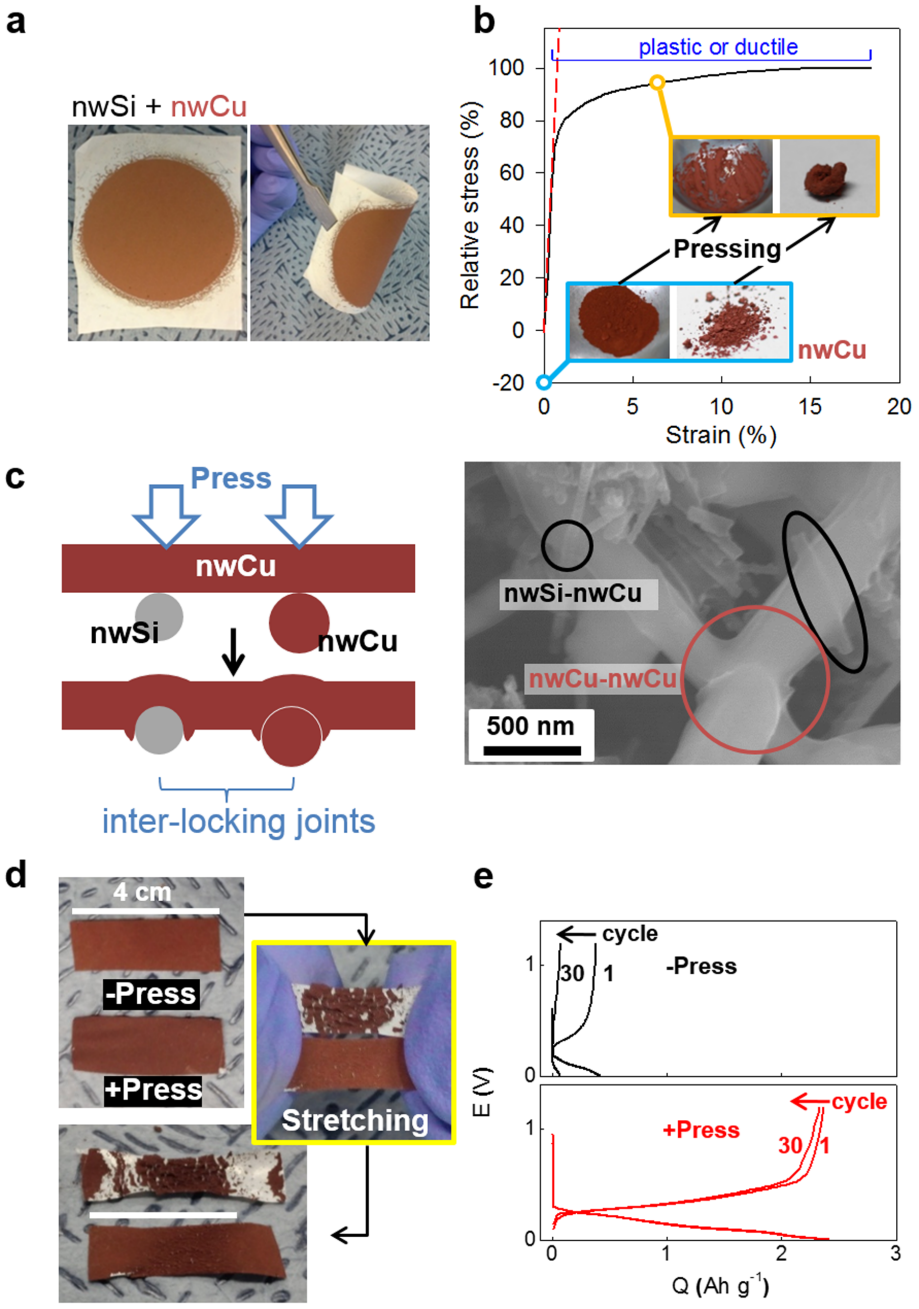
Mechanical properties of SECA. (a) Physical integrity of composites of nwSi with nwCu. (b) Stress-strain curve of copper with snapshots of nwCu before and after pressing. Stress was normalized by its maximum value. (c) Schematic (left) and SEM image (right) image of the structural joints developed between nwSi (or nwCu) and nwCu in an inter-locking way due to ductility of copper. (d) Physical integrity of SECA based on nwCu after stretching. –Press and +Press indicate SECAs before and after pressing at 12 MPa, respectively. (e) Potential profiles of lithiation and delithiation of SECA in a half cell configuration.

**Figure 3:**
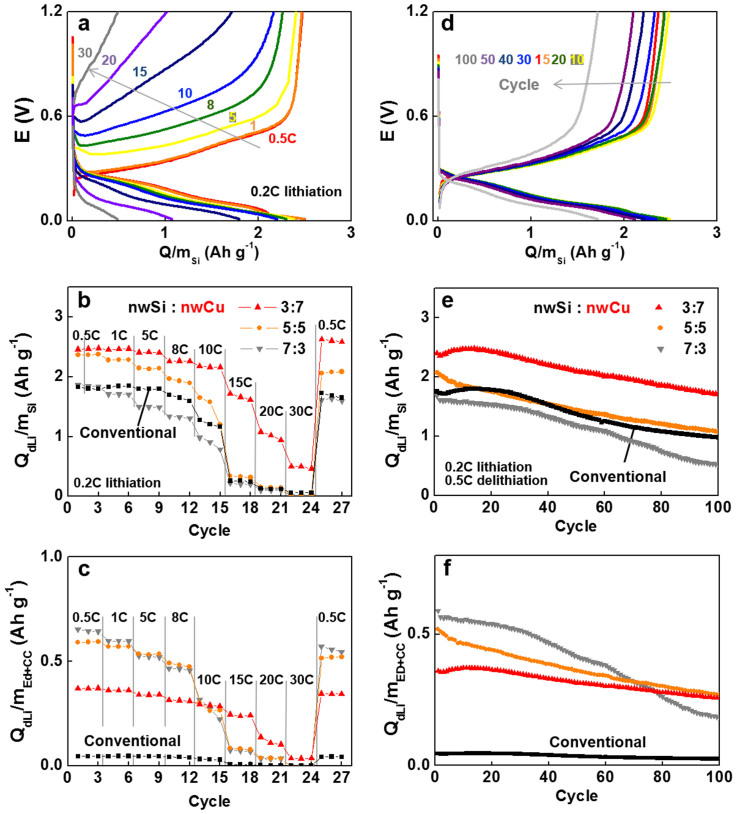
Electrochemical cell performances of SECA (nwSi:nwCu = 3:7) in a half coin cell configuration. (a) Potential profiles at galvanostatic lithiation (0.2C) and delithiation (various C rates as indicated). (b, c) Delithiation rate dependency of capacity. SECA was lithiated at 0.2C. Capacity was normalized by silicon mass (m_Si_) in (b) and by total mass including electrode and current collector (m_Ed + CC_) in (c). (d) Potential profiles traced along cycling lithiation at 0.2C and delithiation at 0.5C. (e, f) Capacity retention of SECAs with three different compositions (nwSi:nwCu indicated) during cycling lithiation at 0.2C and delithiation at 0.5C. Capacity was normalized by the same way as in (b) and (c).

**Figure 4:**
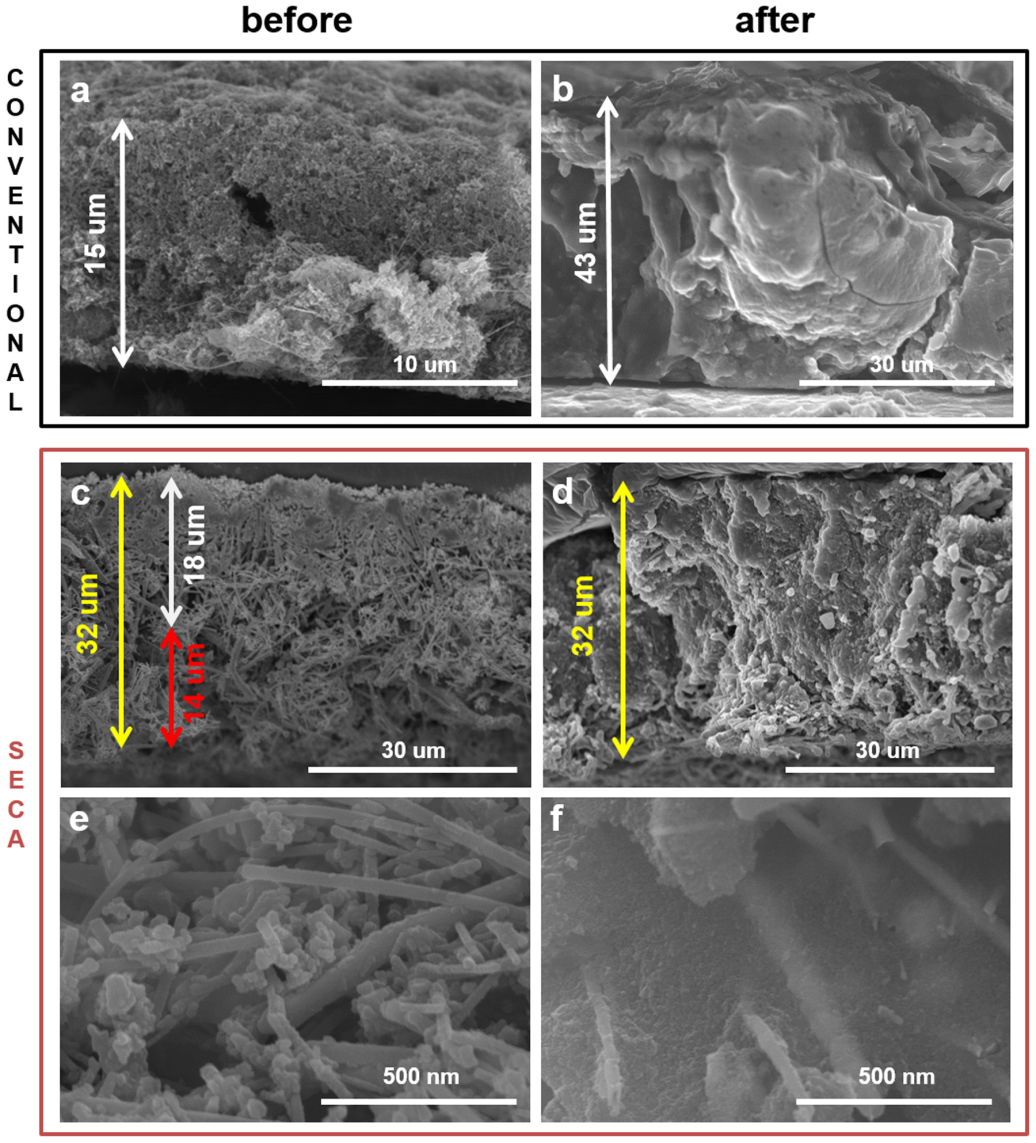
Volume change of the SECA between before and after cycling lithiation/delithiation 100 times. Scanning electron microscopic images of cross-sections of the conventional electrodes (a,b) and the SECA of 30 % nwSi (c–f). The photos in the left and right columns correspond to morphology before and after cycling, respectively. White, red and yellow arrows indicate the thicknesses of electrode, current collector and their assembly, respectively. The weight of silicon loading was fixed at 0.7 mg cm^−2^ for the conventional electrode and the SECA of 30 % nwSi.
