# Supplementary material for: All-in-one assembly based on 3D-intertangled and cross-jointed architectures of Si/Cu 1D-nanowires for lithium ion batteries
Source: Sci Rep. 2015 Feb 27;5:8623. doi: 10.1038/srep08623 (PMC4342559; doi:10.1038/srep08623)
Supplement: Supplementary Information — supporting information [file srep08623-s1.pdf]

Supporting Information

All-in-one assembly based on 3D-intertangled and cross-jointed architectures of Si/Cu 1D-nanowires for lithium ion batteries

*Chihyun Hwang, Tae-Hee Kim, Yoon-Gyo Cho, Jieun Kim and Hyun-Kon Song**

School of Energy and Chemical Engineering, Ulsan National Institute of Science and Technology (UNIST), Ulsan 689-798, Republic of Korea

*The authors to whom correspondence should be addressed.
E-mail: philiphobi@hotmail.com

1. Experimental

Morphological characterization. Cold field emission scanning electron microscopy equipped with energy dispersive X-ray spectrometer (FE-SEM with EDS; Hitachi S-4800) and transmission electron microscopy (TEM; JEOL JEM-2100) were used for morphological characterization. To obtain cross-sectional view of electrodes, electrodes were broken immediately after being immersed in liquid nitrogen.

Electrochemical Characterization. Coin-type half cells (2032R) were assembled in Argon-filled glove box. Electrolyte was 1.3 M $LiPF_6$ in 3:7 (v/v) ethylene carbonate (EC): diethyl carbonate (DEC) with 10% fluoroethylene carbonate (FEC). SECA was used as a working electrode while lithium metal was used as a counter electrode. In a conventional configuration, the separator (polyethylene; Asahi NH716) was sandwiched between the conventional electrode and lithium metal. The cells were galvanostatically charged/discharged in a voltage range of 0.01 V to 1.2 V versus Li^+/Li using a cycle tester (WonATech, WBCS 3000 battery measurement system). The cells were stabilized at 0.5C for the first cycle (1C = 3700 mAh g^{-1}).

2. Calculation

<u>Figure 1d. Electrode mass as a function of silicon loading</u>

Mass of electrodes (m) consists of four components:

$$m = m_A + m_B + m_C + m_{CC}$$

where A = active material (nwSi in this work), B = Binder, C = conducting agent (carbon black in this work) and CC = current collector. All of the four components should be considered with heavy amount of current collector in conventional electrode systems. However, m_B can be neglected in our SECA because nwCu plays roles of binders as well as conducting agents. One more different thing between the conventional electrode and our SECA is m_{CC}: 16 mg cm^{-2} for the conventional versus 1.3 mg cm^{-2} for the SECA. m_{CC} is

independent of the silicon loading (m_A) while m_B and m_C depends on m_A at a fixed composition of electrodes. 6:2:2 A/B/C composition (A = nwSi, B = PVdF and C = Super P) was used for the conventional electrode while three different A/C compositions (A = nwSi and C = nwCu; 3:7, 5:5 and 7:3) were tested for the SECA.

Figure 1e and f. The lines-on-a-plate model for calculating the number of contact points (n_c) and the contact-to-contact distance (L_{cc})

■ **Model description**

Nanowires of the minor component are evenly distributed on the plate consisting of nanowires of the major component.

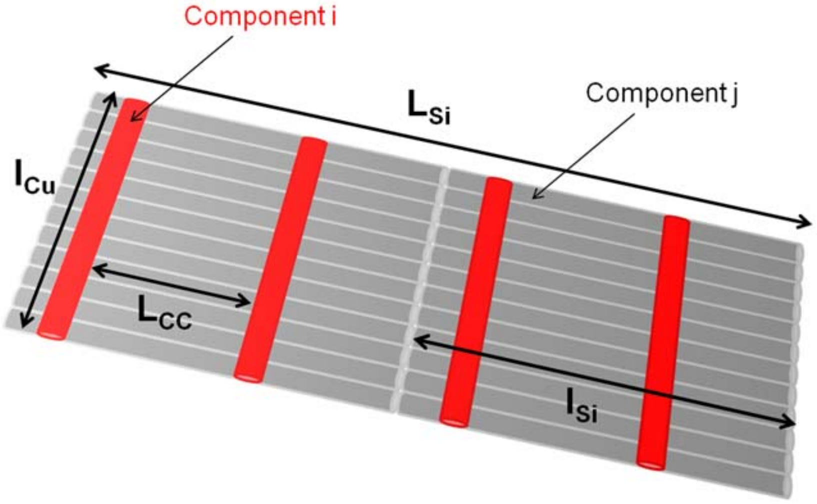

■ **Definition**

1 = nwSi   2 = nwCu

r = radius of nanowires (nm): $r_1 = 20$, $r_2 = 50$

l = length of nanowires

d = density (g cm^{-3}): $d_1 = 2.32$, $d_2 = 8.96$

n = number of nanowires   A = trajectory area of a plate consisting of nanowires

n_c^{max} = maximum values of the number of contact points between nwSi and nwCu

L_{cc}^{min} = minimum values of the contact-to-contact distance

x_1 = nwSi contents (x_{Si}) in nwSi/nwCu composites x_1^{opti} = x_1 to maximize n_c^{max}/m_{tot}

m_{tot} = total electrode mass

■ **Derivation**

- **Mass of each component**

  $m_i = m_{tot} x_i$ and $\sum x_i = 1$

- **Number of each component**

  $$n_i = \frac{m_i}{d_i \times (\pi r_i^2 l_i)}$$

- **Trajectory area of a plate consisting of nanowires**

  $A_i = 2 r_i n_i l_i$

- n_c^{max}

  If $A_i < A_j$ (i = minor & j = major),

  $$n_c^{max} = \frac{l_i}{2r_j} \times n_i = \frac{m_i}{2\pi r_i^2 r_j d_i}$$

  $$\frac{n_c^{max}}{m_{tot}} = \frac{x_i}{2\pi r_i^2 r_j d_i}$$

  which is independent of l_i and m_{tot}.

- L_{cc}^{min}

  For i = 1 and j = 2,

  $$L_{cc}^{min} = 2r_2$$

  $$\frac{L_{cc}^{min}}{2r_1} = \left(\frac{r_2}{r_1}\right)$$

  For i = 2 and j = 1,

  $$L_{cc}^{min} = \frac{l_1^{effective}}{(n_2 - 1)} \cong \frac{l_1^{effective}}{n_2}$$

With

$$l_1^{effective} = \frac{A_1}{l_2} = \frac{2r_1 n_1 l_1}{l_2} = 2r_1\left(\frac{l_1}{l_2}\right) \times \frac{x_1 m_{tot}}{d_1 \pi r_1^2 l_2} \;\&\; n_2 = \frac{m_{tot} x_2}{d_2 \pi r_2^2 l_2}$$

$$\therefore L_{cc}^{min} = 2r_1\left(\frac{x_1}{x_2}\right)\left(\frac{d_2}{d_1}\right)\left(\frac{r_2}{r_1}\right)^2$$

$$\frac{L_{cc}^{min}}{2r_1} = \left(\frac{r_2}{r_1}\right)^2\left(\frac{d_2}{d_1}\right)\left(\frac{x_1}{1-x_1}\right)$$

which is independent of l_i and m_{tot}.

- x_1^{Opti}

At $A_i = A_j$,

$$x_1^{Opti} = \frac{1}{1+\left(\frac{r_2}{r_1}\right)\left(\frac{d_2}{d_1}\right)}$$

At $x_1 = x_1^{Opti}$,

$$\frac{n_c^{max}}{m_{tot}} = \frac{x_1^{Opti}}{2\pi r_1^2 r_2 d_1} = \left(\frac{1}{r_1^3}\right)\frac{x_1^{Opti}}{2\pi d_1\left(\frac{r_2}{r_1}\right)}$$

$$\frac{L_{cc}^{min}}{2r_1} = \left(\frac{r_2}{r_1}\right)$$

The cross-oriented stack model for calculating void fraction

■ Model description

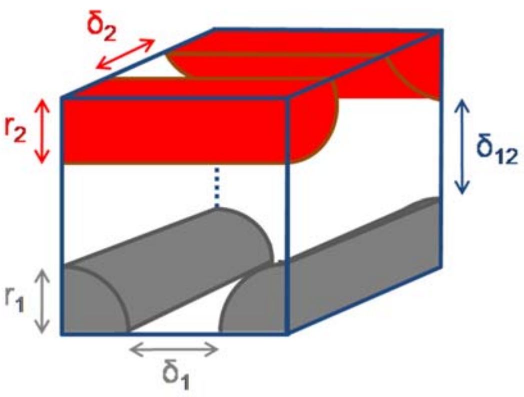

■ Definition

B = binder C = conducting agent (carbon black)

r = radius of nanowires (nm): 20 for nwSi; 50 for nwCu

δ = gap distance V = volume d = density m = mass

■ Derivation

Volume		Mass
$V_{tot} = (2r_1 + \delta_1)(2r_2 + \delta_2)(r_1 + r_2 + \delta_{12})$<br>$V_1 = \frac{1}{4}\pi r_1^2 \times (2r_2 + \delta_2) \times 2$<br>$V_2 = \frac{1}{4}\pi r_2^2 \times (2r_1 + \delta_1) \times 2$	→	$m_1 = V_1 d_1$<br>$m_2 = V_2 d_2$<br>$m_A = m_1 + m_2$
		↓
$V_B = m_B/d_B$, $V_C = m_C/d_C$<br>$V_{void} = V_{tot} – (V_1 + V_2 + V_B + V_C)$<br>Void fraction = $f_{void} = V_{void}/V_{tot}$<br>Volume expansion ratio that can be accommodated by voids = $1 + f_{void} / (1-f_{void})$	←	For the conventional electrode<br>$m_1 : m_B : m_C = \alpha : \beta : \gamma$<br>$m_B = \frac{\beta}{\alpha} m_1$, $m_C = \frac{\gamma}{\alpha} m_1$

○ SECA

1 = nwSi, 2 = nwCu, $V_B = V_C = 0$, $\delta_1 = \delta_{12} = 0$, $\delta_2 = L_{cc} – 2r_2$

○ The conventional electrode

1 = 2 = nwSi, $d_B = 1.78$ g cm^{-3}, $d_C = 1.8$ g cm^{-3}, α = 6, β = 2, γ = 2, $\delta_1 = \delta_2 = \delta_{12}$

Table S1. Comparison between our work and previous works of other groups in terms of the constitution and configuration of electrodes and their electrochemical performances.

	Active material	Conducting Agent	Binder	Current collector	Capacity (C-rate, cycle) (mAh g^{-1})	Coulombic efficiency (cycle) (%)
*Our Work (SECA)	SiNW (nwSi)	CuNW (nwCu)	Not used	CuNW	2500 (0.5C/0.2C, 10th)<br>1900 (0.5C/0.2C, 100th)<br>5C/0.2C > 95 %	65 (1st)<br>99 (> 4th)
Our Work (Conventional)	SiNW	Carbon black (CB)	PAA/CMC	Cu foil	1900 (0.5C/0.2C, 10th)<br>1000 (0.5C/0.2C, 100th)<br>5C/0.2C > 95 %	79 (1st)<br>99(> 4th)
*1	SiNW@C	CNT	Not used	Cu foil	2000 (0.1C/0.1C, 10th)<br>1000 (0.1C/0.1C, 100th)<br>3C/0.1C = 30 %	50 (1st)<br>99 (> 10th)
2	Si grown on nanopaper	Not used	Not used	Conductive nanopaper	1500 (0.1C/0.1C, 10th)<br>1250 (0.1C/0.1C, 100th)<br>1C/1C = 70 %	50 (1st)<br>99 (>20th)
3	SiNW@C	Not used	Not used	200 nm Cu thin film	2000 (0.2C/0.2C, 10th)<br>1500 (0.2C/0.2C, 100th)<br>2C/0.2C = 37 %	88 (1st)<br>99 (>10th)
4	SiNW@C	Not used	Not used	Graphitic carbon	2100 (0.1C/0.1C, 10th)<br>1500 (0.1C/0.1C, 30th)<br>4.5C/0.2C = 30 %	78 (1st)<br>99 (> 20th)
5	SiNW@C	Not used	PAA	Cu foil	2500 (0.1C/0.1C, 10th)<br>2000 (0.1C/0.1C, 100th)<br>1C/0.05C = 25 %	85 (1st)<br>99 (>2nd)
6	SiNT	CB	PVDF	Cu foil	1900 (0.1C/0.1C, 10th)<br>750 (0.1C/0.1C, 90th)	82 (1st)<br>99 (>10th)
6	SiNW	CB	PVDF	Cu foil	1500 (0.1C/0.1C, 10th)<br>300 (0.1C/0.1C, 90th)	87 (1st)<br>99 (>10th)
*7	VNW ($V=V_2O_5$)	CNT gel (25 wt. %)	Not used	Not used	5C/1C = 66 %	-

*Asterisk in the first column indicates that inter-tanglement between active materials and conducting agents are expected in the corresponding electrodes.

NW = nanowires; NT = nanotubes

1 = B. Wang *et al.*, ACS Appl. Mater. Interfaces 5, 6467, (2013)
2 = L. Hu *et al.*, Nano Energy 2, 138, (2013)
3 = W. Wang *et al.*, Nano Energy 2, 943, (2013)
4 = B. Wang *et al.*, Nano Lett. 13, 5578, (2013)
5 = T. D. Bogart *et al.*, ACS Nano 8, 915, (2014)
6 = J.K. Yoo *et al.*, Adv. Mater. 24, 5452, (2012)
7 = X. Jia *et al.*, Energy Environ. Sci. **5**, 6845 (2012).

Table S2. Sheet resistances of current collector

	Before Pressing SECA current collector	After pressing SECA current collector
Sheet resistance (Ω/sq)	Inaccessible State	50

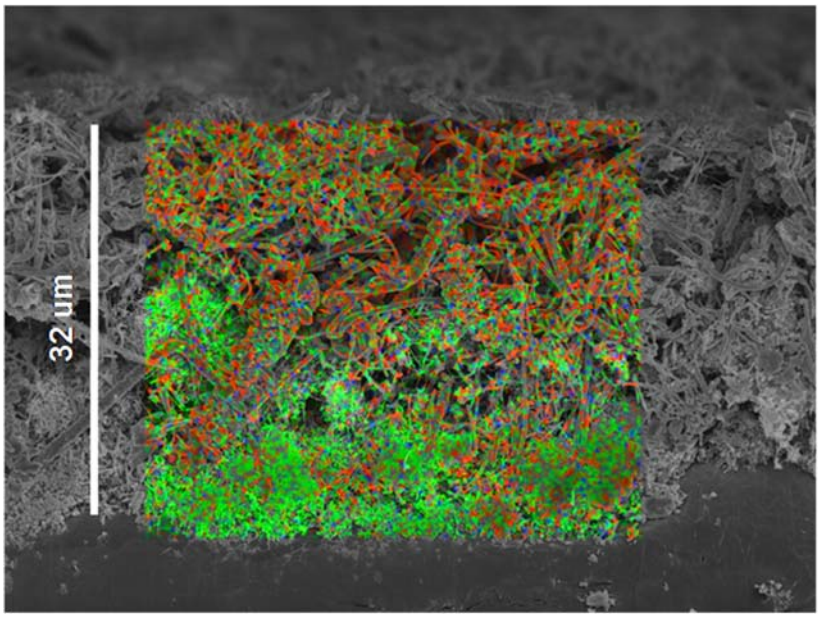

Figure S1. Element mapping of cross-section of SECA with nwSi:nwCu = 3:7 (red = Cu and green = Si) by energy dispersive X-ray spectroscopy (EDS). The red-dominant part (upper) is the current collector layer consisting of nwCu. The relatively green dominant part (lower) indicates the composite layer of nwSi and nwCu.

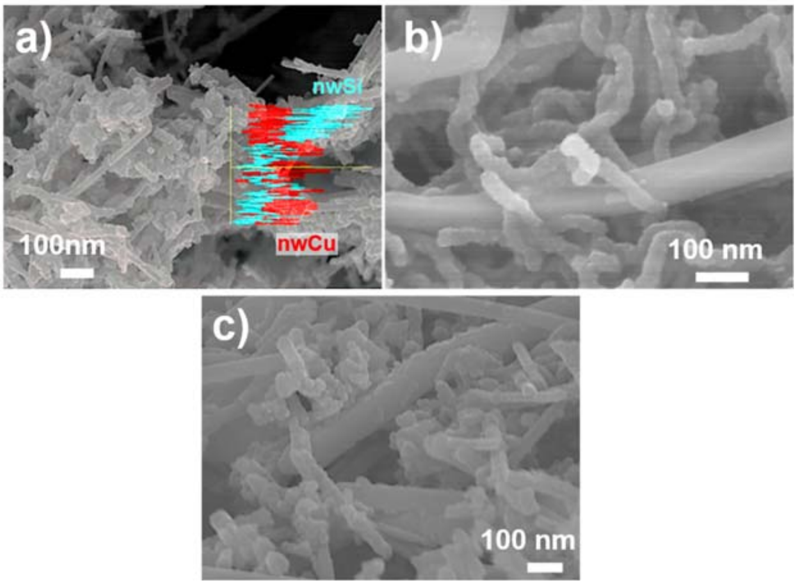

Figure S2. High-resolution cross-sectional views of the SECA by SEM. Line mappings of Cu (red) and Si (blue) by energy dispersive spectroscopy (EDS) were included in (a).

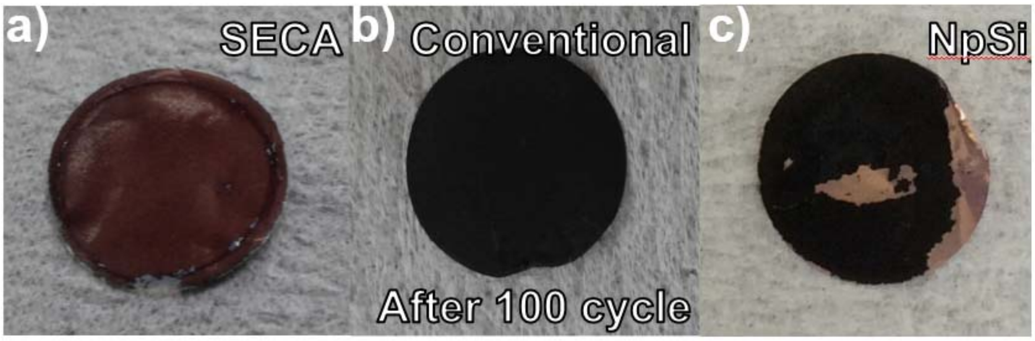

Figure S3. Photos of electrodes after 100 cycles of charge/discharge. (a) SECA. (b) Conventional electrode based on nwSi. (c) Conventional electrode based on silicon nanoparticles (diameter = 50 nm with a spherical shape). The SECA and the conventional electrode based on nwSi showed no crack or fragmentation while the conventional electrode based on silicon nanoparticles was cracked. nwSi demonstrated benefits of good integrity due to its anisotropic dimensionality.

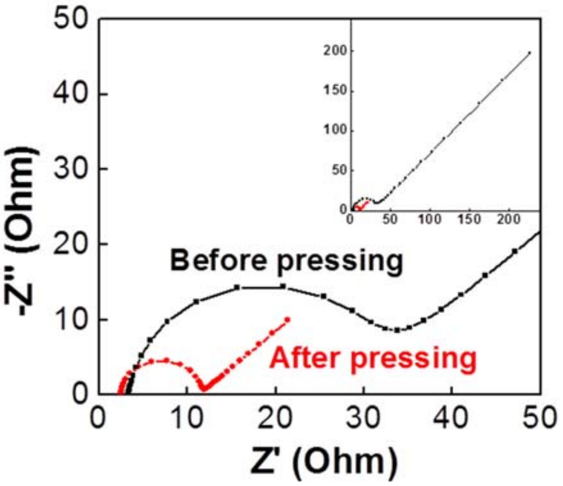

Figure S4. Electrochemical impedance spectra of SECA electrode after and before pressing. The impedance data obtained from a frequency range of 200 kHz to 100 mHz after full lithiation were through the equivalent Randles circuit.

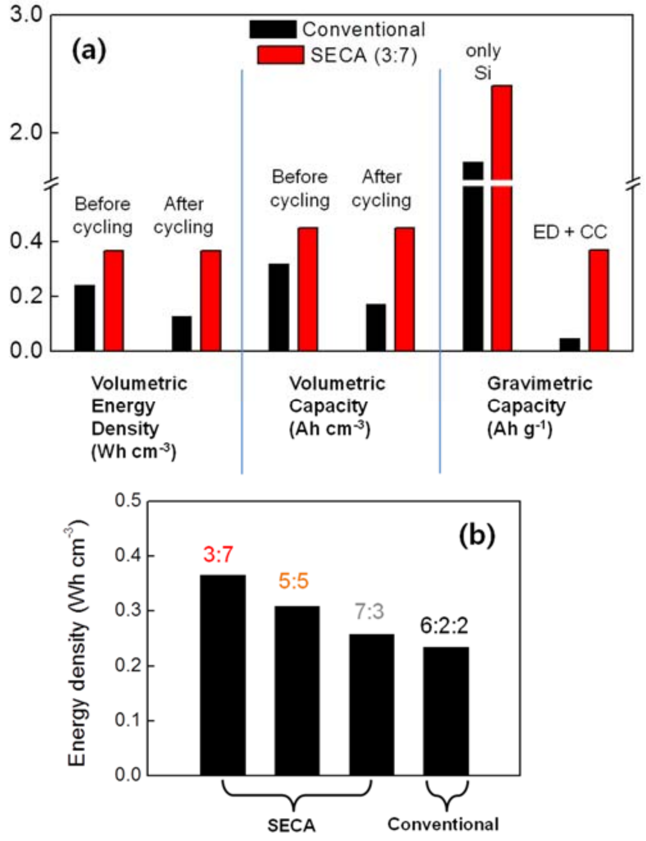

Figure S5. (a) Comparison of energy densities and capacities between our SECA of 30 wt. % nwSi and the conventional tri-component electrodes. The volume expansion experienced after 100 cycles was considered for "After cycling". The third section compares the capacities per silicon mass with those per total mass including electrodes and current collector before cycling. (b) Comparison of volumetric capacities between the conventional control and the

SECA of various nwSi contents. Smaller total volume of electrode is achieved with higher contents of nwCu because density of copper is higher than that of silicon. The same amount of nwSi for all SECA's and even the conventional electrodes were used at 0.6 mg Si cm^{-2} for fair comparison.

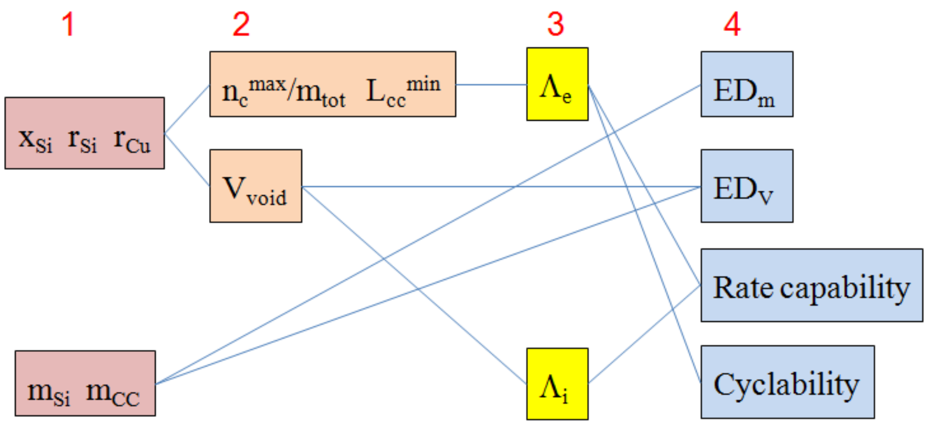

Primary design parameter	• m_{Si} = the amount of nwSi in nwSi/nwCu composites<br>• x_{Si} = composition of nwSi in nwSi/nwCu composites<br>• m_{CC} = the amount of nwCu current collector (CC)<br>• r_{Si} and r_{Cu} = Radii of nwSi and nwCu
Secondary dimensional characteristics	• n_c^{max}/m_{tot} = maximum number of contacts between nwSi and nwCu (n_c^{max}) per total mass (m_{tot}) of the nwSi/nwCu composites<br>• L_{cc}^{min} = minimum contact-to-contact distance<br>• V_{void} = void volume
Tertiary electrochemical characteristics	• Λ_e = Electric conductance<br>• Λ_i = Ionic conductance
Quaternary electrochemical performances	• ED_m = Specific energy density<br>• ED_V = Volumetric energy density<br>• Rate capability

Figure S6. Connectivity between design parameters, dimensional and electrochemical characteristics and resultant performances. Lower-level factors affect higher-level ones. For example, x_{Si} is the factor to control V_{void}, which affects Λ_i, which determine rate capability. Relatively weak connectivity was neglected.

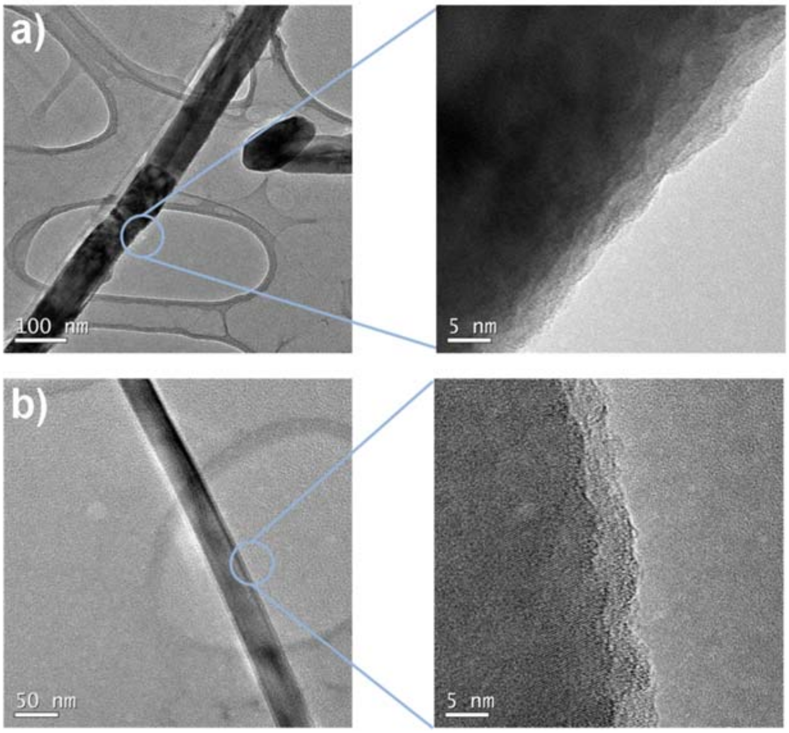

Figure S7. TEM Images of nwCu (a) and nwSi (b)

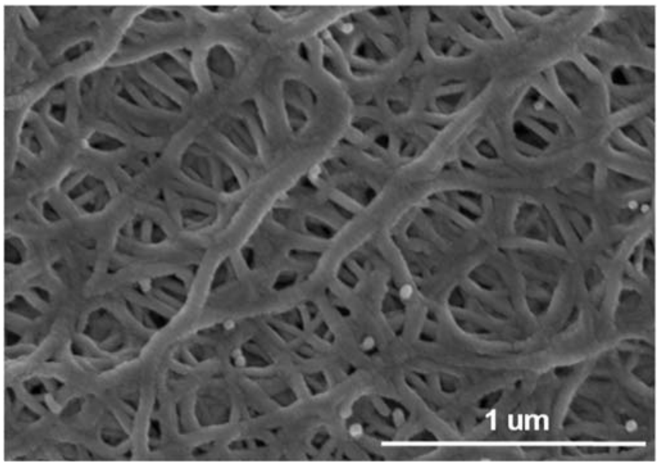

Figure S8. Polymer separator (Asahi NH716)
